# A Novel Heterozygous *IHH* c.331_333del Mutation Identified in a Fetus with Brachydactyly Type A1 Causes IHH Protein Maturation Failure in HEK293T Cells

**DOI:** 10.1007/s43657-024-00191-9

**Published:** 2024-12-21

**Authors:** Ting Zhu, Lijie Guan, Dan Chen, Yi Luo, Mianmian Zhu, Rongyue Sun, Jiamin Shi, Qiu Wang, Yuan Chen, Yihong Wang, Hongwei Wang, Zhongqiu Lu, Dan Wang

**Affiliations:** 1 Department of Pediatrics, The First Affiliated Hospital of Wenzhou Medical University, No. 2 Fuxue Road, Wenzhou, Zhejiang 325000 P. R. China; 2 Zhejiang Key Laboratory of Intelligent Cancer Biomarker Discovery and Translation, First Affiliated Hospital, Wenzhou Medical University, Wenzhou, 325035 China; 3 Department of Ultrasound Imaging, The First Affiliated Hospital of Wenzhou Medical University, Wenzhou, Zhejiang 325000 P. R. China; 4 The Key Laboratory of Emergency and Disaster Medicine of Wenzhou, The First Affiliated Hospital of Wenzhou Medical University, Wenzhou, Zhejiang 325000 P. R. China; 5 Department of Pediatrics, Yongjia People’s Hospital, Wenzhou, Zhejiang 325000 P.R. China; 6 Department of Pediatrics, Taizhou Women and Children’s Hospital of Wenzhou Medical University, Taizhou, Zhejiang 318000 P.R. China

**Keywords:** Indian hedgehog signaling molecule, Brachydactyly type A1, Novel mutation, Fetal short limbs

## Abstract

Brachydactyly A1 (BDA1) is a rare disorder characterized by the disproportionate shortening of fingers and/or toes with or without symphalangism. Mutations in *Indian hedgehog signaling molecule* (*IHH*), which impair the effect of functional IHH protein derived from its precursor IHH, are commonly identified in patients with BDA1 or acrocapitofemoral dysplasia (ACFD). The ultrasound phenotype of fetuses with *IHH* mutations has rarely been described. To better understand the consequences of *IHH* mutation, we analyzed the characteristics of a Chinese fetus with BDA1 caused by a novel heterozygous *IHH* mutation. Clinical data and genomic DNA were collected from the proband and family members. Whole-exome sequencing (WES) was performed to identify potential causative mutations. Sequence analysis was performed to investigate the conservation of the affected leucine residue in IHH. Protein 3D modeling was performed to predict the effects of the mutation on protein structure. In vitro overexpression transfection experiments in human embryonic kidney 293T (HEK293T) cell lines were performed to evaluate the pathogenicity of the identified mutation. The fetal proband carried a novel heterozygous mutation in *IHH* (NM_002181.4: c.331_333delCTG, NP_002172.2: p.Leu111del) inherited from the father; this mutation manifested as shortening of the limbs, with more severe shortening observed in the proximal extremities than in the distal extremities, as evidenced by ultrasound. The Leu111 residue is highly conserved among vertebrates, and deletion of this residue destabilizes the protein structure. Western blotting analysis of HEK293T cells in overexpression transfection experiments revealed that the Leu111del mutation led to an increase in the level of the IHH precursor and a reduction in the level of functional IHH protein compared with those in HEK293T cells expressing wild-type IHH, indicating that this mutation might cause IHH protein dysmaturity. The novel heterozygous mutation c.331_333delCTG (p.Leu111del) in the *IHH* gene is the likely cause of BDA1 in this Chinese fetus. This mutation causes IHH protein maturation failure. These findings contribute to our understanding of the molecular pathogenesis of BDA1 and the clinical identification of fetal BDA1.

## Introduction

Brachydactyly (BD) is a heterogeneous disorder causing abnormal development of the digits, characterized by the disproportionate shortening of fingers and/or toes with or without symphalangism (Temtamy and Aglan 2008). BD can be a simplex malformation or a part of a complex syndrome (Gülen et al. 2010). Simplex BD is divided into seven types (A1, A2, A3, B, C, D and E) according to the pattern of skeletal involvement (Fitch 1979). BDA1 (Brachydactyly A1, OMIM: 112,500) is an extremely rare limb malformation (Gao et al. 2009). The major clinical manifestation is pronounced bilateral brachydactyly affecting the middle phalanges and short stature (Lodder et al. 2008; Jürgen et al. 2018; Vasques et al. 2018). In addition to these manifestations, some patients may also exhibit hearing loss, microcephaly and dactylosymphysis (Salian et al. 2017; Rosettia et al. 2018). BDA1 is mainly diagnosed according to the presence of mutations in specific genes.

Notably, BDA1 is the first disorder recorded to have a mendelian autosomal dominant inheritance pattern in humans (Gao et al. 2009). According to the Online Mendelian Inheritance in Man database, the genes linked to BDA1 are *GDF5* on chromosome 20q11, *BMPR1B* on chromosome 4q22 and *IHH* on chromosome 2q35. Therefore, genetic heterogeneity is an important feature of BDA1 (Degenkolbe et al. 2013).

Among the genes with mutations related to BDA1, *IHH* (OMIM: 600,726), is a key regulator associated with cartilage development and bone formation; the protein is composed of two distinct domains, the N-terminal HH-signal domain (IHH-N) and the C-terminal Hint domain (Pereira et al. 2014). In IHH-producing cells, the IHH protein is first synthesized as a 46 kDa precursor in the endoplasmic reticulum. This precursor undergoes autocatalysis, producing the functional 20 kDa IHH-N protein (Gang et al. 2011; Huan et al. 2022). IHH-N binds to Patched (Ptc), rescuing the inhibition of Smoothened (Smo) by Ptc. Smo is then translocated to the cilium via autocholesterylation, where it upregulates target genes, including *Ptc1*,*Hip1*, and *Gli1*, in the hedgehog signaling pathway (Vasques et al. 2018; Ingham 2022; Yuhong and Jiang 2022; Ao et al. 2022). It has been demonstrated that the mutations E95K and D100E enhance the degradation of IHH-N via the lysosomal pathway and impair IHH-PTC binding, further affecting the hedgehog signaling pathway (Gang et al. 2011).

Here, we investigated the clinical and genetic characteristics of BDA1 in a Chinese fetus and identified a novel *IHH* deletion mutation, c.331_333delCTG (p.Leu111del), that might be associated with BDA1. We also evaluated the pathogenicity of this novel mutation by using bioinformatics tools and in vitro cellular functional verification and summarized the *IHH* mutations related to BDA1. Our findings expand the known *IHH* mutation spectrum associated with BDA1, which improves our understanding of the genetic basis of this disease from a clinical perspective and provides guidance for molecular diagnosis and genetic counseling.

## Materials and Methods

### Participants

The proband and family members were enrolled at the First Affiliated Hospital of Wenzhou Medical University in May 2022. Written informed consent was obtained from the family of the proband before the beginning of the study. Our study was approved by the Ethics Committee of the First Affiliated Hospital of Wenzhou Medical University (Ethics approval number: KY2022-R177).

### Ultrasound Examination

Abdominal ultrasound scans were performed with the GE Voluson E10 ultrasound system using the C1 − 6 and RM7C abdominal ultrasound probe models, with probe frequencies of 2 − 5 MHz and 2 − 8 MHz, respectively. According to the guidelines issued by Hadlock (Hadlock et al. 1991) and Jeanty (Jeanty et al. 1982), the biparietal diameter (BPD), head circumference (HC), abdominal circumference (AC), femoral neck (FL), tibia (TIB), fibula (FIB), humerus (HL), radius (RAD) and ulna were measured and evaluated.

### Whole-Exome Sequencing (WES) and Sanger Sequencing

WES of the family was performed. We extracted genomic DNA from fetal amniotic fluid (30–40 mL) and peripheral blood (3–5 mL) from the parents with a Qiagen DNeasy Blood & Tissue Kit (Qiagen, Hilden, Germany) according to the manufacturer’s instructions. Exome enrichment was performed using an Agilent SureSelect Human All Exome V6 Kit (Agilent Technologies, Santa Clara, CA, USA) following the manufacturer’s protocol. Then, the products were sequenced on a NovaSeq 6000 sequencer (Illumina, San Diego, CA, USA) with a total sequence length of 34,962 Mbp and a sequencing depth of 149×. The quality of the raw sequencing data was assessed using fastp software (Chen et al. 2018), and low-quality reads and adaptor-contaminated reads were discarded. The filtered data were then aligned to the human hg19 reference genome using Burrows–Wheeler Aligner software. Subsequently, the capture efficacy was assessed. The Genome Analysis Toolkit was used to identify single-nucleotide mutations and insertions and deletions in the genome. The identified SNVs and indels from this family were then screened against the OMIM, HGMD and ClinVar population databases. Based on the American College of Medical Genetics and Genomics (ACMG) guidelines for the evaluation of genetic mutations (Bahcall 2015), the mutations were categorized as pathogenic (P), likely pathogenic (LP), variant of uncertain significance (VUS), likely benign (LB), or benign (B). Sanger sequencing was performed to verify suspected mutations in the family (Korneluk et al. 1985).

### Conservation Analysis and Pathogenic Assessment

Sequence conservation of the IHH protein was examined using the ClustalW and SnapGene programs and PhastCons. The potential pathogenicity of gene mutations on protein function was assessed using the Franklin (https://franklin.genoox.com/), Rare Disease Data Center (https://www.rdgta.org.cn/) and MutationTaster (https://www.mutationtaster.org/) websites. SWISS_MODEL (https://swissmodel.expasy.org/) and PyMOL were used to predict the three-dimensional structure of the wild-type (PDB number: 3K7G) and mutant proteins.

### Plasmid Construction, Cell Culture, Transfection and Western Blotting Analysis

Plasmids expressing Flag-IHH-Wt and FLAG-IHH-Mu with the full coding sequence of IHH (GenBank: NM_002181.4) were obtained from Miaoling Bioscience & Technology (Wuhan, China). All constructs were confirmed through sequencing. HEK293T cells were obtained from the American Type Culture Collection (Washington, DC, USA) and cultured at 37 °C in a 5% CO_2_ atmosphere in Dulbecco’s modified eagle medium supplemented with 10% fetal bovine serum and 1% penicillin‒streptomycin. Subsequently, the cells were transfected with 2.5 µg of plasmid using Lipofectamine 3000 (Thermo Fisher Scientific, Waltham, MA, USA) for 48 h before further use. An empty pCMV vector was used as the control. Transfected HEK293T cells were harvested and lysed in RIPA buffer supplemented with protease and phosphatase inhibitors. Protein concentrations were determined using a bicinchoninic acid protein assay kit. The proteins were then separated on 10% polyacrylamide gels and transferred to polyvinylidene fluoride membranes using electroblotting. Next, the membranes were blocked with 5% milk in Tris-buffered saline containing Tween-20 for 1 h and then incubated overnight at 4 °C with anti-FLAG (diluted 1:1000, Cat# 14,793, Cell Signaling Technology, Danvers, MA, USA) and anti-GAPDH (diluted 1:10,000, Cat# 10494-1-AP, Proteintech, Danvers, MA, USA) primary antibodies. The target proteins were detected using secondary goat anti-rabbit antibodies (diluted 1:8,000, BL003A, Biosharp, Hefei, China). Subsequently, the immunoblots were visualized using the ECL Plus Western blotting substrate and scanned with a Typhoon FLA 9500 (GE Healthcare Life Sciences, Fairfield, MI, USA).

### Statistical Analysis

Statistical analysis was conducted using GraphPad Prism 9.3 (San Diego, CA, USA). The results are expressed as the mean ± standard deviation (SD). Two independent-sample *t* tests were employed to assess statistical significance, which was indicated by *p* < 0.05.

## Results

### Clinical Manifestations

**Fig. 1:**
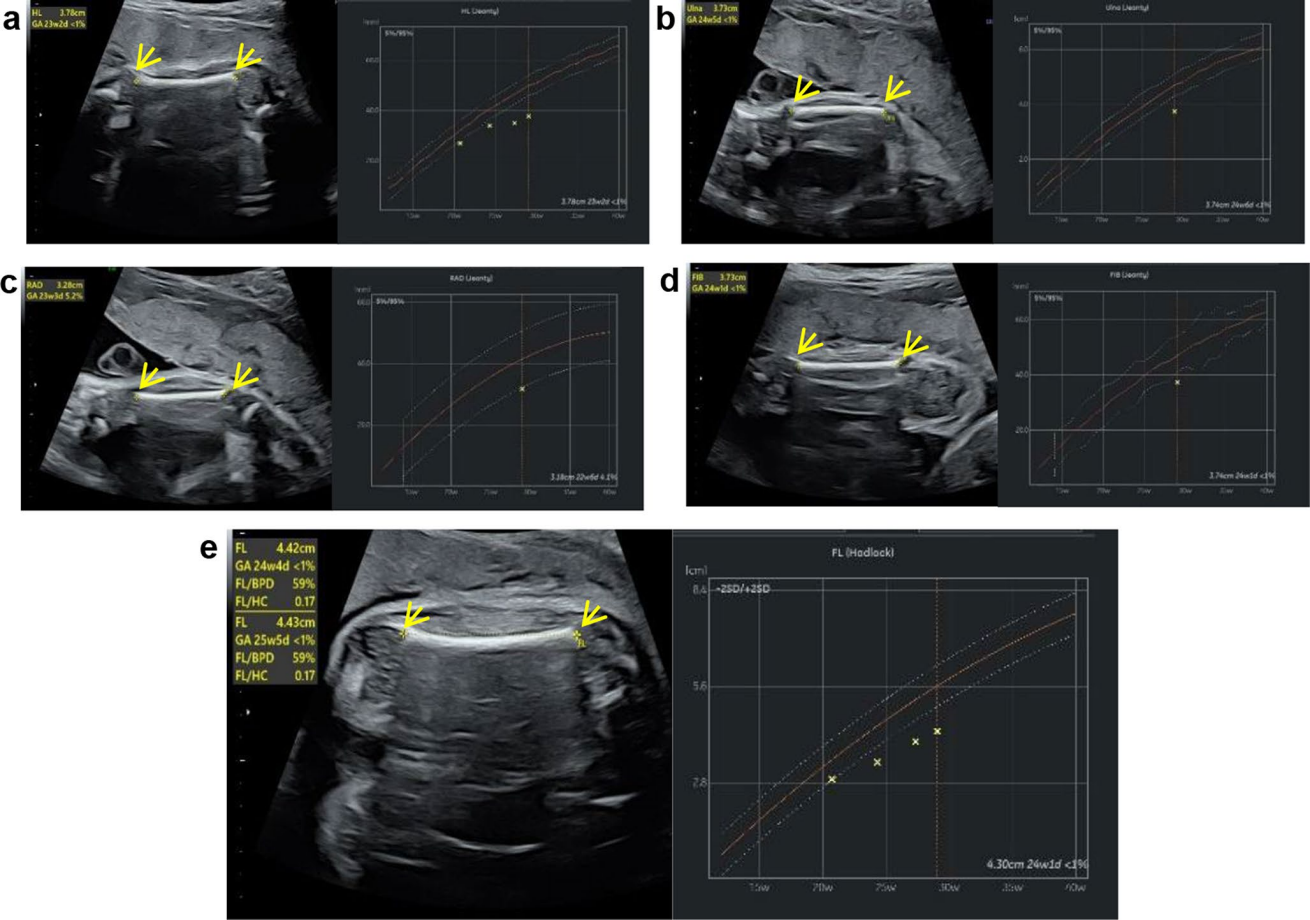
Ultrasound scan of the proband revealing the short limbs. **a** Humerus length (HL). **b** Ulna. **c** Radius (RAD). **d** Fibula length (FIB). **e** Femur length (FL)

**Fig. 2:**
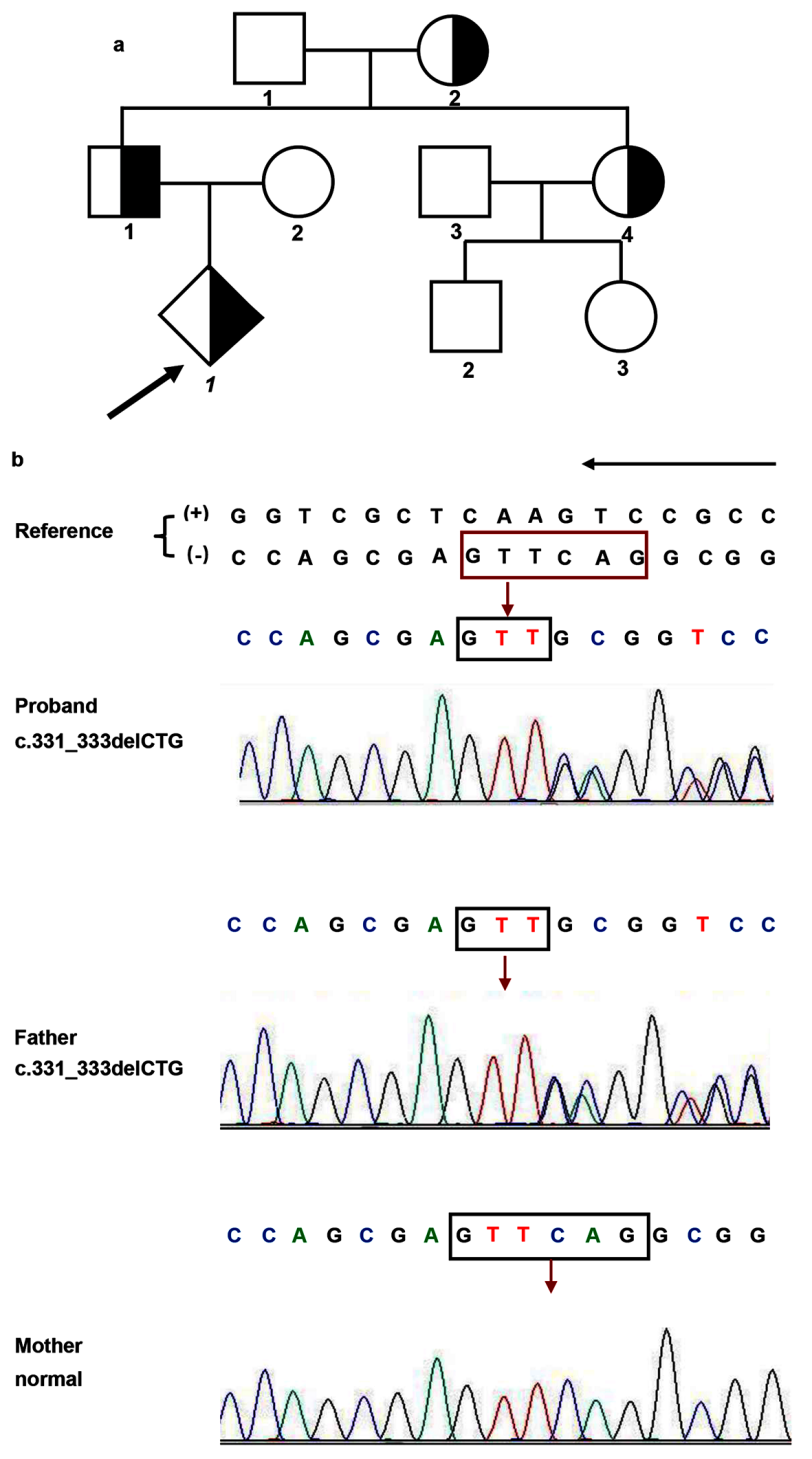
Diagram of the pedigree and Sanger sequencing. **a** Pedigree of a Chinese family with autosomal dominant BDA1. Squares indicate males, circles indicate females, and rhombuses indicate relatives of unknown sex. The filled half-black symbols indicate individuals with disease phenotypes. The proband is indicated with an arrow. **b** Sanger sequencing showing the *IHH* 331_333delCTG mutation. The red arrows indicate mutation sites. The black arrow indicates the forward orientation of the reference sequence. (+): coding strand of the reference sequence, (-): template strand of the reference sequence. Variations are represented based on the coding strand

A pregnant 22-year-old woman was seen at our hospital for routine prenatal ultrasonography in May 2022. Ultrasonography performed at 29^+ 3^ weeks from the last menstrual period indicated a gestational age of 27^+ 4^ weeks and further revealed obvious short limb deformities in the fetal proband. Specifically, the HL was 3.78 cm (− 5.0 SD) (Fig. 1a), the ulna length was 3.74 cm (− 3.9 SD) (Fig. 1b), the RAD length was 3.18 cm (− 1.7 SD) (Fig. 1c), the TIB length was 3.79 cm (− 4.2 SD), the FIB length was 3.74 cm (− 2.6 SD) (Fig. 1d), and the FL length was 4.30 cm (− 4.4 SD) (Fig. 1e) (Table 1). The maniphalanxes were not discernible. Shortening of the proximal extremities was more severe than that of the distal extremities. To explore a potential genetic cause for the observed short limbs of the fetus, we performed a pedigree survey, which revealed that some family members had brachydactyly and short stature. The grandmother (I-2) had a height of 142 cm (− 2 SD), and an aunt (II-4) had a height of 145 cm (− 2 SD); furthermore, the father (II-1) had a height of 164 cm (− 1 SD), which was within the lower normal range. Unfortunately, we did not obtain images or radiographs of the relevant fingers of the family members. These findings suggested that the phenotype of the proband likely had a hereditary origin (Fig. 2a).

**Table 1:** Ultrasound data of the proband at a gestational age of 29^+ 3^ weeks

Measurements	Value	Deviation
BPD (Hadlock)	7.47 cm	0.5 SD
HC (Hadlock)	26.41 cm	−1.1 SD
AC (Hadlock)	24.71 cm	−0.2 SD
FL (Hadlock)	4.30 cm	−4.4 SD
TIB (Jeanty)	3.79 cm	−4.2 SD
FIB (Jeanty)	3.74 cm	−2.6 SD
HL (Jeanty)	3.78 cm	−5.0SD
RAD (Jeanty)	3.18 cm	−1.7 SD
Ulna (Jeanty)	3.74 cm	−3.9 SD

BPD: biparietal diameter; HC: head circumference; AC: abdominal circumference; FL: femoral neck; TIB: tibia; FIB: fibula; HL: humerus; RAD: radius; SD: standard deviation

### Genetic Findings

**Fig. 3:**
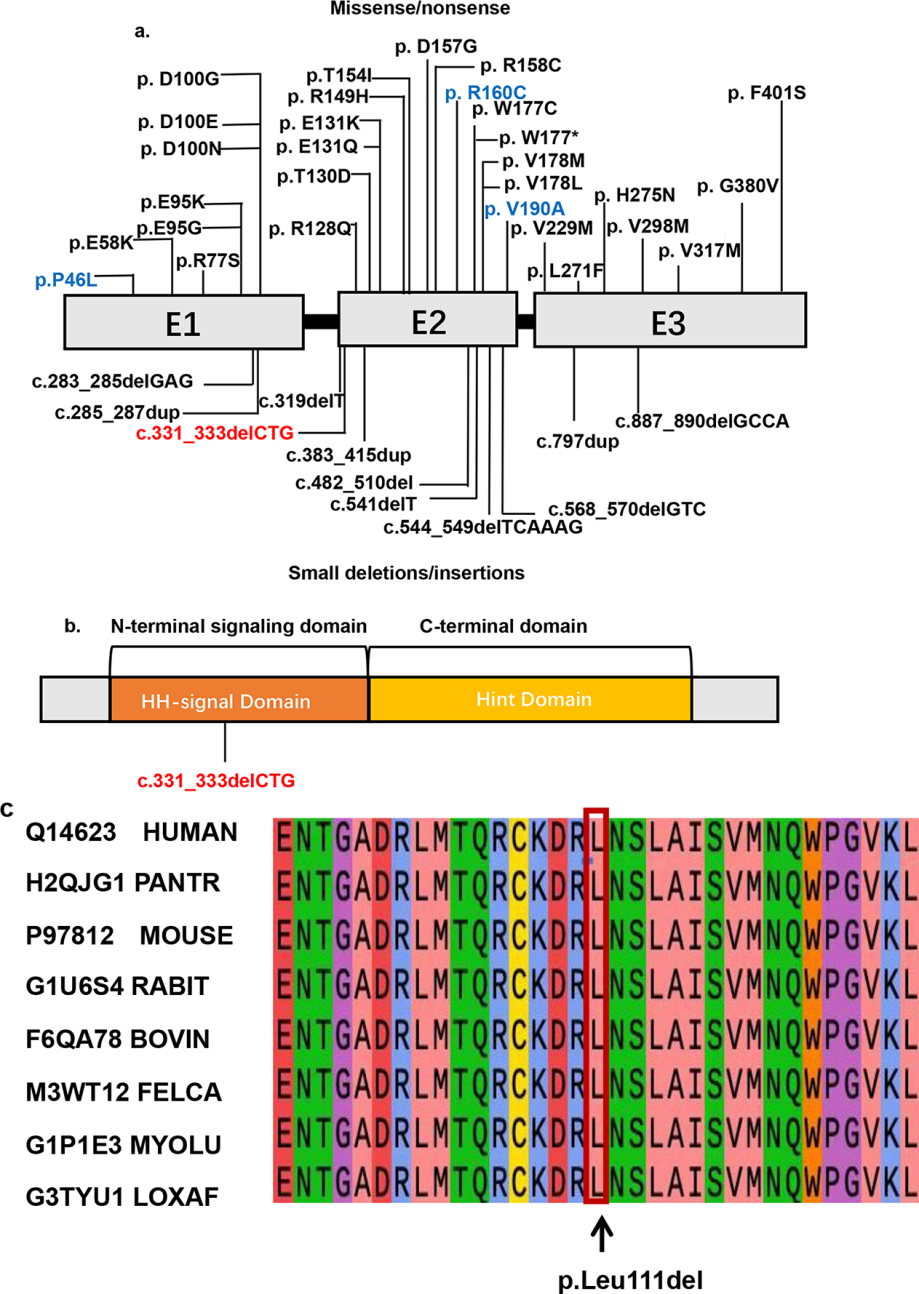
Localization of disease-associated variations in *IHH*. **a** Schematic representation of *IHH* gene mutations; gray boxes represent three different exons (E1: exon 1, E2: exon 2, and E3: exon 3), and black boxes represent the introns of the *IHH* gene. Vertical lines indicate the positions of missense/nonsense (above the boxes) and small deletion/insertion (below the boxes) mutations. Blue indicates homozygous mutations in ACFD. **b** Key domains of the IHH protein. N-terminal HH-signal domain (IHH-N) and the C-terminal Hint domain. p.Leu111 is located in the middle of the HH signal domain. **c** Conservation of the p.Leu111del mutation across various species

To identify potential mutations responsible for the hereditary short stature phenotype in this family, we performed WES and Sanger sequencing on blood samples collected from the parents and an amniocentesis sample from the proband, revealing a rare heterozygous mutation in the *IHH* gene in the proband (Fig. 2b). The mutation was a three-base deletion at complementary DNA positions 331 to 333 (c.331_333delCTG) in exon 2 of *IHH* (Fig. 3a) in the proband and father (II-1). Unfortunately, we were unable to obtain the genotypes of the aunt and grandmother due to sample limitations. This deletion was predicted to cause a loss of the leucine residue at position 111 (p.Leu111del) in the IHH-N of the IHH protein (Fig. 3b) and was classified as VUS (PM2 or PM4) according to the ACMG guidelines (Bahcall 2015). Notably, this mutation has not been reported previously in the 1000 Genomes Project, the Exome Aggregation Consortium, or the Genome Aggregation Database. These findings indicate that this mutation is likely the causative mutation. In addition, no other variants were found.

### Pathogenic Assessment and Conservation Analysis

The pathogenicity of c.331_333delCTG in the *IHH* gene was evaluated using several methods and was predicted to be VUS or LP (Table 2). This mutation, c.331_333delCTG (p.Leu111del), is located in the HH-signal domain of the IHH protein (Fig. 3b), which is important for all patterning activities (Ingham 2022). Crucially, a comparison of IHH sequences from various mammals, including humans (UniProt: Q14623), chimpanzees (UniProt: H2QJG1), mice (UniProt: P97812), rabbits (UniProt: G1U6S4), bovines (UniProt: F6QA78), cats (UniProt: M3WT12), little brown bats (UniProt: G1P1E3), and African elephants (UniProt: G3TYU1), revealed that amino acid 111 of IHH is 100% conserved across these species. Moreover, the conservation score of Leu111 in PhastCons was one, indicating that Leu111 is highly conserved (Fig. 3C). These data suggest that L111 is key to the normal biological function of the IHH protein.

**Table 2:** Pathogenicity prediction and conservation analysis of the mutations

Gene	IHH
Chromosome	Chr2:219919146–219,925,643
Nucleotide	c.331_333delCTG
Amino Acid	p.Leu111del
Franklin	VUS
RDDC	LP
Mutation Taster	DC
PhastCons 100 way score	1 (highly conserved)

LP: likely pathogenic; VUS: uncertain significance. RDDC: rare disease database. DC: disease causing. The chromosome position is based on GRCh37/hg19

### Effect on 3D Protein Structure

**Fig. 4:**
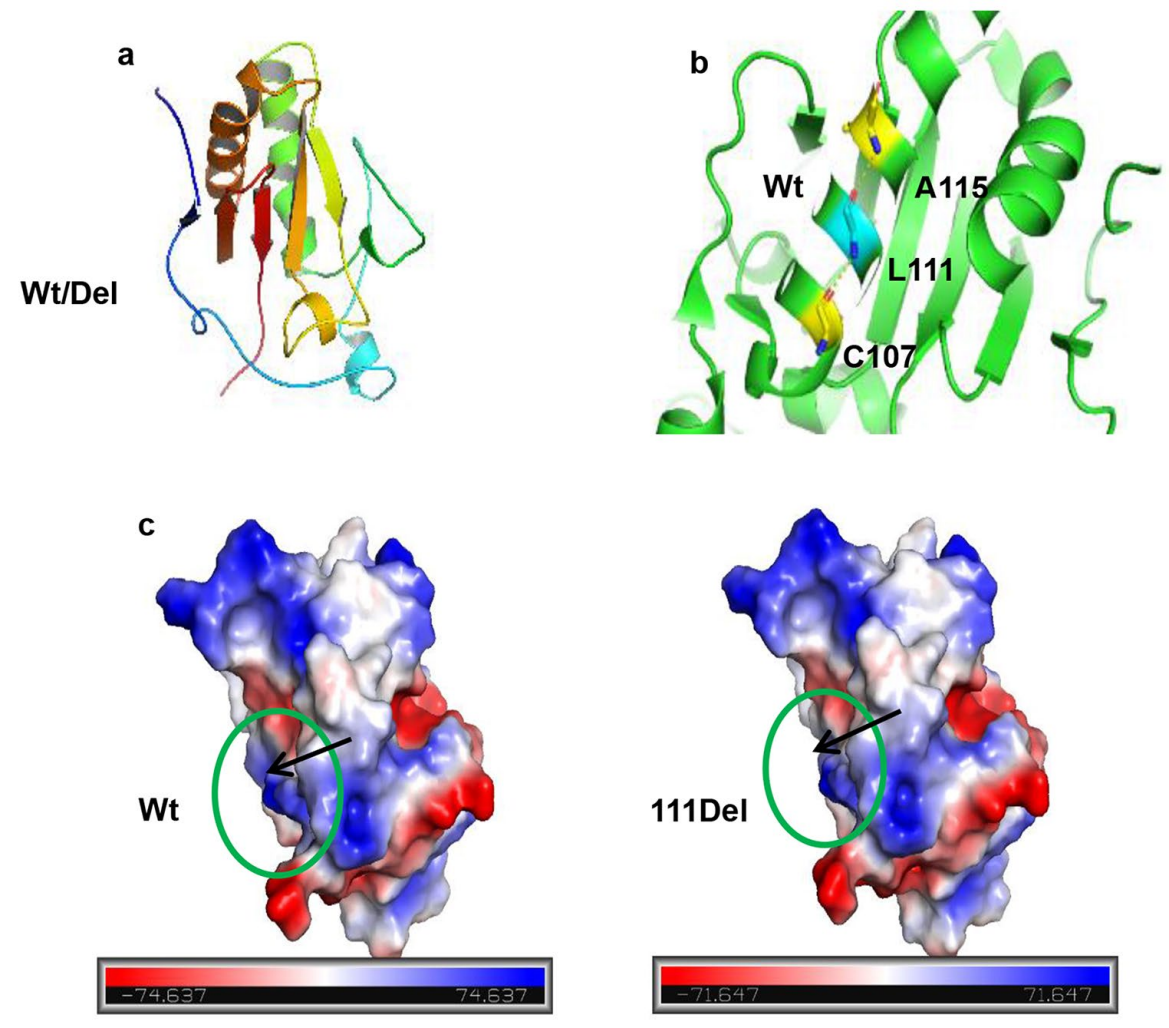
Crystal structure and electrostatic potentials of the human IHH-N protein. **a** Schematic representation of the human IHH-N protein. **b** Location and hydrogen bonding of Leu111 in the Wt human IHH-N protein. **c** Electrostatic potentials of the human IHH-N protein. Compared with that of the Wt protein, the 111del mutation altered the electrostatic potential at the protein surface. The structure of the 111del mutation also showed high flexibility, as indicated by the black arrow. The green circle indicates the positive pocket near the 111del mutation. The electrostatic potentials of the protein surface were calculated using PyMOL. Wt: wild-type. A: alanine, L:leucine, C:cystine

To investigate the effect of the mutation on protein structure, we modeled the structures of the mutant and wild-type IHH-N proteins using SWISS-MODEL, as described in the methods section. Both the wild-type and mutant structures included an α + β core with two α-helices, a six-stranded β-sheet core with a two-stranded antiparallel β-sheet, and three short helices (Fig. 4a). The p.Leu111del mutation is located in the HH-signal domain and results in the absence of the nonpolar aliphatic amino acid Leu, which has a long side chain. This leucine residue was predicted to participate in the interior stabilization of the α-helices of the IHH protein and to form hydrogen bonds with C107 and A115 (Fig. 4b). Given that the mutation was near the positively charged ligand-binding pocket (Fig. 4c), the electrostatic potential of the protein’s surface decreased in the mutant protein, which could reduce the affinity of the IHH protein for its ligand. Moreover, the predicted structure of the mutant protein showed high flexibility (Fig. 4c). Based on these observations, we hypothesize that the *IHH* (c.331_333delCTG) (p.Leu111del) mutation might destabilize the interior structure of the IHH protein and reduce its ligand-binding ability, thereby impacting its stability and function.

### IHH-N Levels were Decreased in HEK293T Cells Expressing IHH p.Leu111del

**Fig. 5:**
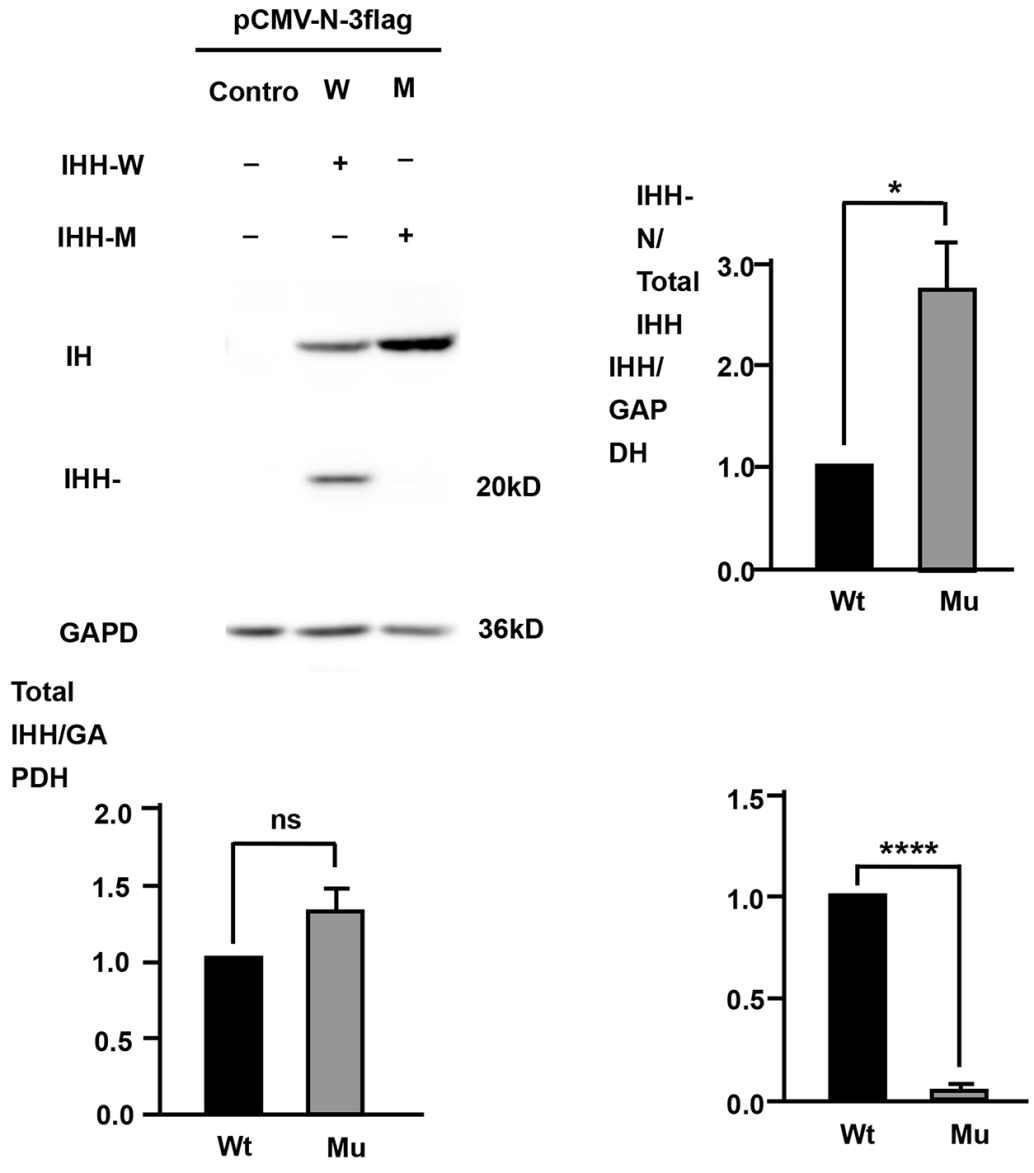
The c.331_333delCTG mutation inhibits IHH precursor maturation to IHH-N. HEK293T cells were transfected with plasmids bearing FLAG-tagged wild-type (Wt, lane 1) or mutant (Mu, lane 2) IHH, and cell extracts were analyzed by western blotting with anti-FLAG (Cat. #14,793, Cell Signaling Technology). The molecular masses (kDa) are indicated on the right; the precursor IHH protein is 46 kDa, and the IHH-N protein is 20 kDa. Cells transfected with an empty vector were used as controls. All data are shown as the mean ± SD of three independent experiments. Statistically significant differences are denoted by asterisks (*) with a significance level of *p < 0.05 and ****p < 0.0001. Total IHH: total IHH + IHH-N

To assess the effect of c.331_333delCTG on IHH protein expression, we analyzed HEK293T cells transfected with expression plasmids encoding FLAG-tagged wild-type (Wt) or mutant (Mu) IHH. Western blotting analysis of the whole-cell extracts showed that the level of the precursor IHH was markedly elevated in cells transfected with the p.Leu111del construct compared to that in cells transfected with the Wt construct, the expression of the IHH-N fragment almost disappeared in cells transfected with the Mu protein, and the total IHH + IHH-N was similar for both constructs (Fig. 5). The IHH-N fragment is generated by cleavage of the signal peptide from the precursor IHH via self-catalysis. Therefore, we hypothesize that the c.331_333delCTG mutation may cause dysmaturity of the IHH protein.

## Discussion

Brachydactyly type A1 caused by *IHH* mutation is an autosomal dominant disorder. In this study, we investigated a fetus with short limb deformities that could be attributable to a heterozygous mutation in the *IHH* gene. WES of the proband and his family members revealed a novel *IHH* c.331_333delCTG (p.Leu111del) mutation inherited from the affected father. Computational analysis and western blotting analysis demonstrated that this deletion causes the failure of IHH protein maturation in HEK293T cells. According to the ACMG guidelines, c.331_333delCTG in *IHH* was classified as VUS (Bahcall 2015). However, based on our bioinformatic analysis and in vitro cellular results, *IHH* c.331_333delCTG (p.Leu111del) appears to be deleterious and could be reclassified as LP. Thus, our study suggested that the *IHH* c.331_333delCTG (p.Leu111del) mutation may have caused the disease in this family by disturbing IHH maturation.

Our structural analyses indicated that the missing leucine residue, which has a long side chain, was located in an α-helix and was a part of the super secondary structure αβα. These data suggested that this nonpolar leucine residue participates in the interaction with the β-sheet. Additionally, *IHH* c.331_333delCTG (p.Leu111del) is positioned near previously identified pathogenic mutation hot spots in *IHH* (codons 95, 100 and 131) and may affect the structure of this region in a similar way (Gang et al. 2011). Previous molecular dynamics simulation experiments showed that all pathogenic mutations located in IHH-N could affect the structure and biological function of the IHH protein, decreasing the total mean potential energy and reducing the calcium and zinc cofactor binding energies (Vasques et al. 2018). However, additional basic experimental analyses are needed to confirm these models.

The IHH protein is a secretory protein produced by prehypertrophic chondrocytes. After translation, the IHH precursor is transformed into the functional IHH-N signaling molecule by autocatalytic cleavage and subsequent modification with cholesterol and palmitate. In our study, the expression of the IHH-N fragment carrying the p.Leu111del mutation almost completely disappeared, and the expression of the related precursor IHH markedly increased. Therefore, we speculate that the c.331_333delCTG mutation may cause dysmaturity of the IHH protein.

*IHH* is related to the formation and differentiation of cartilage and bones (Huan et al. 2022). The diseases related to small *IHH* mutations are BDA1 and acrocapitofemoral dysplasia (ACFD), a rare autosomal recessive skeletal dysplasia. To date, 40 pathogenic *IHH* mutations in 48 probands have been reported in the HGMD and ClinVar database (Fig. 3b; Table 3). Among these patients, the ultrasound phenotype of fetuses with *IHH* mutations has rarely been described. Our study enriches the phenotype spectrum of IHH mutations. A previous study reported that BDA1-related mutations are confined to the central region of the N-terminal fragment of *IHH*, whereas ACFD-related mutations are located at the distal N- and C-terminal regions of *IHH* (Byrnes et al. 2009). However, in recent studies, heterozygous mutations have been detected throughout the *IHH* gene (Fig. 3b). Compared to BDA1, ACFD is rarer and has a more severe phenotype characterized by short postnatal-onset disproportionate stature with short limb dwarfism, brachydactyly and a narrow thorax (Geert et al. 2019) (Table 3). In animal models, compared to heterozygous IHH^+/*E95K*^ mice, homozygous IHH^*E95K*/*E95K*^ mice exhibit a classic BDA1 phenotype with much shorter or even absent middle phalanges (Gao et al. 2009). The clinical manifestations of the homozygous c.478 C > T (p.Arg160Cys) mutation are much more serious than those of the heterozygous mutation (Pelin and Mehmet 2021). Furthermore, monoallelic dominant-negative mutations in *IHH* lead to BDA1, and biallelic loss-of-function mutations cause ACFD. In our study, short limbs with more severe shortening in the proximal extremities than in the distal extremities were the most significant feature. It is difficult to distinguish BDA1 from ACFD on the basis of clinical manifestations, and these findings implied the potential convergence of BDA1 and ACFD. The observed phenotypic variations may be contingent upon the expression levels of the IHH protein.

**Table 3:** Summary of pathogenic *IHH* mutations and the clinical presentation of BDA1 and ACFD

	Mutation	Patient ethnic origin	Age (years)	Height (SD)	Diagnosis	Imaging findings	Affected family members (Height SDs)	Reference
1	c.172G > A p.Glu58Lys	Brazil	3.4	−2.8	het-BDA1	Cone-shaped epiphyses of middle phalanx of second and fifth fingers and distal phalanx of thumb	Mother − 2.20, Uncles − 4.30, − 4.30, − 2.20, Aunt − 3.30, Cousins − 3.10, − 2.20, − 1.90, − 1.70, − 1.50, − 0.80	(Vasques et al. 2018)
2	c.228_229delinsAA p.Arg77Ser	Spain	13.0	−2.2	het-BDA1	Shortening of the middle phalanges and metacarpals of the hand and bilateral metatarsals of the feet	Father − 2.30	(Sentchordi-Montané et al. 2020)
3	c.283_285delGAG p.Glu95del	Spain	11.0	−0.7	het-BDA1	Shortening of all phalanges and metacarpals of the hand	Father − 0.80	(Sentchordi-Montané et al. 2020)
		Netherlands	0.4	−2.0	het-BDA1	Shortening of middle phalanges and proximal phalanges in the thumb	NA	(Lodder et al. 2008)
4	c.283G > A p.Glu95Lys	China	NA	NA	het-BDA1	Middle phalanges missing or fused to the distal phalanges of the hand	NA	(Gao et al. 2001)
5	c.284 A > G p.Glu95Gly	Mexico	NA	NA	het-BDA1	Short stature	NA	(Kirkpatrick and Au 2003)
6	c.285_287dupGAA p.Glu95_Asp96insLys	America	31.0	NA	het-BDA1	Shortening of all digits, missing middle phalanges of the hand	NA	(Rosettia et al. 2018)
7	c.298G > A p.Asp100Asn	New Zealand	35.0	−1.0	het-BDA1	Absent middle phalanges in digits two to five in both the hands and feet and shortened proximal phalanges	NA	(Byrnes et al. 2009)
		India	0.4	normal	het-BDA1	Absence of the middle phalanges	NA	(Salian et al. 2017)
		China	NA	NA	het-BDA1	Broadening and shortening of all digits	NA	(Zhu and Ke 2007)
8	c.299 A > G p.Asp100Gly	China	30.0	−2.4	het-BDA1	Absence of middle phalanges of the fifth finger, absence of middle phalanges of the third to fifth toes, fusion of middle and terminal phalanges of third to fifth toes Radial deviation of the second finger, bilateral shortening of metacarpals 3–5 and metatarsals	Uncle − 2.40 Cousin − 2.50	(Qi et al. 2020)
9	c.300 C > A p.Asp100Glu	China	NA	−1.0	het-BDA1	Short stature	NA	(Gao et al. 2001)
10	c.319delT p.Cys107Alafs*5	Spain	13.0	−2.6	het-BDA1	Brachymetacarpia with shortening of middle phalanx of second and fifth fingers	Mother − 3.30	(Vasques et al. 2018)
11	c.331_333del p.Leu111del	China	GA: 27^+ 4^ week	NA	het-BDA1	Short limb deformities with more severe shortening of the proximal extremities	Grandmother − 2.00, Aunt − 2.00, Father − 1.00	This paper
12	c.383G > A p.Arg128Gln	America	43.0	Normal	het-BDA1	Tarsal coalition and short arms with normal height	NA	(Byrnes et al. 2009)
13	c.383_415dup p.Arg128_His138dup	China	45.0	−0.9	het-BDA1	Shortening of middle phalanx and distal phalanx	Father − 1.18, Daughter + 0.93	(Feier et al. 2022)
14	c.389 C > A p.Thr130Asp	India	NA	NA	het-BDA1	Scoliosis and clubfoot	NA	(Byrnes et al. 2009)
15	c.391G > C p.Glu131Gln	Spain	11.5	0.4	het-BDA1	Shortening of middle phalanx, distal phalanx, metacarpal, and 4th metatarsal	Mother − 2.70	(Sentchordi-Montané et al. 2020)
16	c.391G > A p.Glu131Lys	China	NA	NA	het-BDA1	Middle phalanges missing or fused to the distal phalanges and short stature	NA	(Gao et al. 2001)
		Germany	34.0	NA	het-BDA1	NA	NA	(Byrnes et al. 2009)
		Korean	32.0	Normal	het-BDA1	Absence of middle phalanges and hypoplastic proximal phalanges in both hands and feet	NA	(Mi-Ae et al. 2015)
17	c.446G > A p.Arg149His	Spain	11.0	−1.7	het-BDA1	Shortening of middle phalanx and distal phalanx of the hand, shortening of bilateral metatarsals and phalanges of feet	Father − 3.50	(Sentchordi-Montané et al. 2020)
		Brazil	8.0	−2.6	het-BDA1	Shortening of middle phalanges of second and fifth fingers with cone-shaped epiphyses	Uncles − 2.40, − 1.70	(Vasques et al. 2018)
18	c.461 C > T p.Thr154Ile	China	NA	Normal	het-BDA1	Bilateral shortening of the middle phalanges of the digits, fusion of the middle and distal phalanges of digit 5, unaffected proximal phalanges of digit 1 in both hands	NA	(Liu and Wang 2006)
19	c.470 A > G p.Asp157Gly	Spain	9.5	−0.4	het-BDA1	Shortening of middle phalanges, distal phalanges and metacarpals of the hand and bilateral shortening of the metatarsals of the feet	Father − 4.70	(Sentchordi-Montané et al. 2020)
20	c.472 C > T p.Arg158Cys	Sweden	57.0	normal	het-BDA1	Shortening of middle phalanges and proximal phalanges, hypoplasia of the ulna minus and ulnar styloid processes, osteoarthritis, normal length of all distal phalanges and shortening or absence of the middle phalanges	NA	(Stattin et al. 2009)
21	c.482_510del p.Asn161Serfs*6	Portugal	1.5	−3.8	het-BDA1	Clinodactyly (5th) of the hand	Father − 2.10	(Sentchordi-Montané et al. 2020)
22	c.531G > A p.Trp177*	Spain	11.3	−1.8	het-BDA1	Shortening of middle and distal phalanges and cone-shaped epiphyses of the hand	Mother − 2.60	(Sentchordi-Montané et al. 2020)
23	c.531G > C p.Trp177Cys	NA	16.0	−2.9	Not Available	Severe shortening of middle phalanges of the second and fifth fingers with cone-shaped epiphyses of the hand	Father − 3.70	(Vasques et al. 2018)
24	c.532G > A p.Val178 Met	Brazil	9.4	−4.3	het-BDA1	Shortening of the middle phalanx of the fifth finger	Father − 2.20	(Vasques et al. 2018)
25	c.532G > C p.Val178Leu	Brazil	7.1	−2.8	het-BDA1	No abnormal findings	NA	(Vasques et al. 2018)
26	c.541del p.Glu181Serfs*43	Brazil	2.4	−2.0	het-BDA1	Short hands, broad and short metacarpals of the hand, and cone-shaped epiphyses in the proximal phalanges of the toes	Father − 2.60	(Sentchordi-Montané et al. 2020)
27	c.544_549delTCAAAG p.Ser182Lys183del	Japan	9.0	−2.8	het-BDA1	Shortening of the middle phalanges of the second to fifth digits and fusion of the middle and terminal phalanges. No abnormal findings in either the proximal phalanges or metacarpals of the proband or his father	Siblings − 1.70, − 1.80 Father − 2.20 Aunt − 3.00 Cousin − 2.20	(Nozomu et al. 2021)
28	c.568_570delGTC p.Val190del	Spain	11.5	−2.0	het-BDA1	Shortening of middle and distal phalanges	NA	(Sentchordi-Montané et al. 2020)
29	c.685G > A p.Val229Met	Brazil	12.9	−3.8	het-BDA1	No abnormal findings	Father − 2.20	(Sentchordi-Montané et al. 2020)
30	c.797dupC p.Arg267Thrfs*15	NA	7.0	−3.5	het-BDA1	Severe shortening of the middle phalanges of the second and fifth fingers	NA	(Vasques et al. 2018)
31	c.811 C > T p.Leu271Phe	NA	NA	−2.1	het-BDA1	Short stature	Father − 1.20 Mother − 2.80	(Hauer et al. 2018)
32	c.823 C > A p.His275Asn	Spain	7.3	−2.7	het-BDA1	Shortening of the middle phalanges	Mother − 1.20, Grandfather − 4.00	(Sentchordi-Montané et al. 2020)
33	c.887_890delGCCA p.Ser296Thrfs*68	Spain	5.5	−3.3	het-BDA1	Short hands	Mother − 3.30	(Sentchordi-Montané et al. 2020)
34	c.892G > A p.Val298Met	Spain	17.0	−2.1	het-BDA1	Shortening of middle phalanx and cone-shaped epiphyses	Mother − 2.00, Sister − 2.80, Nephew − 1.70, Aunt − 2.90, Cousin − 2.20	(Sentchordi-Montané et al. 2020)
		Spain	12.0	−3.2	het-BDA1	Shortening of the middle phalanges and clinodactyly and shortening of the bilateral metatarsals	NA	(Sentchordi-Montané et al. 2020)
35	c.949G > A p.Val317Met	Brazil	7.5	−2.6	het-BDA1	Shortening of the middle phalanges	NA	(Sentchordi-Montané et al. 2020)
		Spain	NA	−1.8	het-BDA1	Severe shortening of middle phalanx of fifth finger and slight shortening of middle phalanx of the second finger	Mother − 1.80	(Vasques et al. 2018)
36	c.1139G > T p.Gly380Val	Brazil	9.2	−2.8	het-BDA1	Short stature	NA	(Vasques et al. 2018)
37	c.1202T > C p.Phe401Ser	Spain	7.8	−4.0	het-BDA1	Short stature	Sister − 2.70	(Vasques et al. 2018)
38	c.137 C > T p.Pro46Leu	Belgium	10.5	<−2.0	hom-ACFD	Hand phenotypes resembling those in BDA1; hand X-ray of carrier parent showing no obvious differences. Mild shortening of middle phalanges of carrier parent observed when measured	NA	(Hellemans and Coucke 2003)
39	c.478 C > T p.Arg160Cys	Turkey	28.0	−8.9	hom-ACFD	Fusion of the middle and distal phalanges in the fifth toes, radial angulation, extremely short femoral neck	Father − 1.81, Mother − 3.08, Sisters − 7.30, −3.00	(Pelin and Mehmet 2021)
40	c.569T > C p.Val190Ala	Netherlands	NA	NA	hom-ACFD	Generalized brachydactyly (all tubular bones involved). Carrier parents noted to have relative shortening of metacarpals and proximal phalanges	NA	(Hellemans and Coucke 2003)

NA: not available; het: heterozygote; hom: homozygote; GA: gestational age

We report for the first time the clinical phenotype of a fetus with a heterozygous *IHH* mutation, providing a theoretical basis for the early identification of congenital short limbs and enabling the possibility of early clinical intervention for affected patients (Vasques et al. 2018).

Our data suggested that the novel heterozygous *IHH* c.331_333del mutation causes IHH protein maturation failure in HEK293T cells. Due to sample limitations, we only conducted wild-type and mutant plasmid overexpression experiments in HEK293T cells. The extraction and analysis of cell cultures from patients or the transfection of plasmids into semi-knockdown cell lines would be more effective in simulating the in vivo environment, producing results closer to those in the internal environment. However, based on the clinical presentation of the proband and our analysis, we believe that this did not affect our results.

In conclusion, in a Chinese family, we identified a new heterozygous mutation, c.331_333delCTG (p.Leu111del), in *IHH* that may cause BDA1. Bioinformatics analysis indicated that this deletion mutation was likely deleterious, and western blotting analysis revealed that this mutation markedly decreased the level of the IHH-N protein compared to that of the wild-type protein and increased the expression of the IHH precursor. Thus, our investigations suggested that the *IHH* c.331_333delCTG (p.Leu111del) mutation may have caused the disease in this family by disturbing IHH maturation. Our initial identification of fetal clinical phenotypes paves the way for potential early clinical interventions. These findings contribute to our understanding of the molecular pathogenesis of BDA1 and the clinical identification and intervention of fetal BDA1.

## Conclusion

This study demonstrated that the IHH c.331_333del mutation causes impaired maturation of the IHH protein in HEK293T cells, leading to the development of BDA1. Additionally, this is the first report of the fetal phenotype associated with an IHH mutation, which includes limb shortening, with the proximal extremities being more severely affected than the distal extremities. These findings have potential value in early clinical identification and intervention.

## Data Availability

The data supporting the findings of this study are available from the corresponding author upon reasonable request.

## Abbreviations

BDA1: Brachydactyly type A1
ACFD: Acrocapitofemoral dysplasia
WES: Whole-exome sequencing
IHH: Indian hedgehog signaling molecule
IHH-N: N-terminal HH-signal domain
Ptc: Patched
Smo: Smoothened
BPD: Biparietal diameter
HC: Head circumference
AC: Abdominal circumference
FL: Femoral neck
TIB: Tibia
FIB: Fibula
HL: Humerus
RAD: Radius
VUS: Variant of uncertain significance
LP: Likely pathogenic
ACMG: American College of Medical Genetics and Genomics
Leu: Leucine
HEK293T: Human embryonic kidney 293T cells
SD: Standard deviation
Wt: Wild-type
Mu: Mutant
